# Lessons from the Implementation of Pilot Practices to Tackle the Burden of Noncommunicable Diseases in Europe

**DOI:** 10.3390/ijerph17134661

**Published:** 2020-06-29

**Authors:** Antonio Sarría-Santamera, Lorena Pinilla-Navas, Patricia González-Soriano, Iñaki Imaz-Iglesia, Teresa Moreno-Casbas, Teresa Corral

**Affiliations:** 1 School of Medicine, Nazarbayev University, Astana 020000, Kazakhstan; 2 REDISSEC, 28029 Madrid, Spain; imaz@isciii.es; 3 Global Health Research Group IMIENS-UNED, 28029 Madrid, Spain; mmoreno@isciii.es; 4 Institute of Health Carlos III, 28029 Madrid, Spain; l.pinillanavas@isciii.es (L.P.-N.); pat.gs.92@gmail.com (P.G.-S.); tcorral@isciii.es (T.C.); 5 CIBERFES, 28029 Madrid, Spain

**Keywords:** health plan implementations, chronic disease, evaluation

## Abstract

(1) Background: The gap between research findings and their application in routine practice implies that patients and populations are not benefiting from the investment in scientific research. The objective of this work is to describe the process and main lessons obtained from the pilot practices and recommendation that have been implemented by CHRODIS-PLUS partner organizations; (2) Methods: CHRODIS-PLUS is a Joint Action funded by the European Union Health Programme that continues the work of Joint Action CHRODIS-JA. CHRODIS-PLUS has developed an Implementation Strategy that is being tested to implement innovative practices and recommendations in four main areas of action: health promotion and disease prevention, multimorbidity, fostering quality of care of patients with chronic diseases, and employment and chronic conditions; (3) Results: The Three-Stages CHRODIS-PLUS Implementation Strategy, based on a Local Implementation Working Group, has demonstrated that it can be applied for interventions and in situations and contexts of great diversity, reflecting both its validity and generalizability; (4) Conclusions: Implementation has to recognize the social dynamics associated with implementation, ensuring sympathy toward the culture and values that underpin these processes, which is a key differentiation from more linear improvement approaches.

## 1. Introduction

The gap between research findings and their application in routine practice is well documented. Although there is an obvious necessity to further spread effective innovation across different healthcare settings, significant challenges limit this extension. The emergence of evidence-based healthcare, which led to efforts in applying scientific rigor in the development of high-quality and valid healthcare guidelines, has not been properly followed by the same intensity in the implementation of those evidence-based guidelines. This gap between the generation of knowledge and its application implies that patients and populations are not benefiting from the investment in scientific research [1].

Public health is about changing systems by using an evidence base, capturing the underlying mechanisms that explain why changes occur to allow replication within and across public health environments. Accelerating the adoption of evidence-based programs, practices, and policies has to be a primary goal of public health.

Given the growing burden that noncommunicable diseases (NCD) represent for European countries, this gap between research and practice is unacceptable. The burden of NCD demands not just a scientific community committed to providing the best evidence, but also the feasibility of its application in practice. Knowledge generated through classical typical research projects conducted under ideal conditions or in otherwise highly controlled environments makes it difficult to translate resulting innovations into routine care, taking into account the many factors, and their great diversity, that are often present in studies of public health intervention.

The objective of this work is to describe the process and main lessons obtained from the pilot practices and recommendations that have been implemented by CHRODIS-PLUS partner organizations.

## 2. Materials and Methods

CHRODIS-PLUS is a Joint Action funded by the European Union Health Programme that continues the work of Joint Action CHRODIS [2]. Through CHRODIS-PLUS 42, partners representing 21 European countries collaborate to implement pilot projects and generate practical lessons in the field of NCD supporting member states to reduce the burden of NCD, increase the sustainability of health systems, and develop human capital. CHRODIS-PLUS has four main areas of action:

1. Health promotion and disease prevention;
2. Multimorbidity;
3. Fostering quality of care of patients with chronic diseases;
4. Employment and chronic conditions.

CHRODIS-PLUS partners developed a Three-Stage Implementation Strategy: Pre-Implementation, Implementation, and Post-Implementation, based on the collaborative work among owners of practices, implementers, experts, and local stakeholders, working together conforming a Local Implementation Working Group (LIWG).

## 3. Results

Papers included in this Special Issue provide useful lessons to those with concern, interest, and responsibility to overcome the challenges of the implementation of good practices to address the burden of NCD. The main finding is that CHRODIS-PLUS Implementation Strategy has demonstrated that it can be applied for interventions and in situations and contexts of great diversity, reflecting both its validity and generalizability.

The work by Barnfield and colleagues [3] discusses the results from the cross-country implementation of a series of health promotion interventions for specific population groups in specific settings: children’s health, adults at work, and older adults, exploring the contextual success factors, and barriers for their uptake. In that paper, they describe the challenges that represent the transfer of practices that have been demonstrated to be successful in other contexts. They indicate that implementation is not a straightforward process. Elements that facilitated the implementation were the need for an amount of conformity between current activities and the proposed health promotion programs, the maximization of communication between good practice owners and implementers, and assurance of the participation of key decision-makers.

Palmer et al. [4] report the process for the pilot implementation of the Integrated Multimorbidity Care Model (IMCM) in five sites: two in Spain (the Andalusian Health System and the Aragon Health System), two in Lithuania (Vilnius University Hospital Santaros Klinikos, and Hospital of Lithuanian University of Health Sciences Kauno Klinikos), and one in Italy (Università Cattolica del Sacro Cuore). Their paper explains the use of the Three-Step Implementation Strategy and describes both how the five LIWGs have adapted and applied that methodology for local implementation as well as how those five sites are adapting the IMCM to their local contexts. IMCM was developed by JA-CHRODIS and comprises 16 multidimensional components to improve the care of persons with multimorbidity [5].

In their paper, Zaletel et al. [6] describe the process of development as well as the pilot implementation of the JA CHRODIS Recommendations and Quality Criteria in Finland, Slovenia, Croatia, Serbia, Greece, Germany, Bulgaria, and Spain. Each implementation site is applying the Recommendations and Quality Criteria with a specific purpose and aim. The diversity of settings, domains, and health care organizations is extraordinarily valuable for testing the transferability of the Recommendations and Quality Criteria, identifying key enablers and barriers for its implementation as well as to develop a guide for their future use.

The development of innovative solutions to offer practical solutions for the employment and working conditions of people with NCD is discussed in the paper of Silvaggi et al. [7]. This paper describes the process followed to map the actions that workplaces have taken to support employees’ wellbeing and health, and the work participation of individuals with NCD. The tools presented in their paper provide valuable empirical perspectives to facilitate the development of more feasible, attractive, and effective interventions to foster occupational wellbeing and health benefits both for employees with NCD and employers.

## 4. Discussion

CHRODIS-PLUS is contributing to the alleviation of the growing burden that NCD represents for European countries bridging the unacceptable gap between research and practice. The burden of NCD demands not just a scientific community committed to providing the best evidence, but also the feasibility of its application in practice. Knowledge generated through classical and typical research projects that happen under ideal conditions or in otherwise highly controlled environments hinders the translation of resulting innovations into routine care, taking account of the many and seemingly uncontrollable factors that are often present in public health intervention studies [8].

The types of questions that implementers have to respond to in order to speed up the transfer of research to practice are very different from classical researchers. CHRODIS-PLUS is answering questions like: How relevant is the evidence developed for the contextual reality of other environments? Can interventions that have been demonstrated to be successful in a given environment work out when they move to another practice, system, or policy environment [9]?

Implementation science is the study of methods used to promote the systematic uptake of research into routine practice and to improve outcomes and quality [10], examining the processes that promote the use of well-researched interventions in “real-world” settings.

Implementation science is different from traditional research. Implementation aims to examine factors to enhance the use and sustainability of innovative solutions, rather than analyze intervention outcomes [11], recognizing the complex influences of real-world (policy, organizations, and intended users of a particular intervention). Collaboration and communication between producers of knowledge and stakeholders-users is a central tenant of implementation to adopt and integrate innovations in routine practice [12].

Implementation is challenging: it requires time, commitment, and mutual interests. Scientific knowledge focusing on intervention outcomes, based on mechanistic and lineal perspectives [13], without considering its application in real-world settings, would not succeed in its adequate implementation. Interventions must be outlined with their intended users in mind, considering the potential barriers to implementation, including resources, knowledge, skills, and the competing demands on providers. For promoting the uptake of research in practice, it is necessary to adapt the intervention to fit the diverse array of local contexts [14].

The frameworks that have been developed to categorize can be summarized into three main categories [15]:

(a) Describing and/or guiding the process of translating research into practice;
(b) Understanding and/or explaining what influences implementation outcomes; and
(c) Evaluating implementation efforts.

The process of implementation is deeply connected with complexity. Complexity science has been utilized to conceptualize the implementation and translation of evidence into practice [13]. Complexity science challenges the conventional wisdom that because an effect was observed in a well-controlled experiment it will occur similarly and with the same effects in other situations. In complexity science, the components of a system, namely, the agents and their artifacts, are important, but secondary to the relationships between these components [16]: agents communicate and learn from each other and their environment, adjusting their behavior accordingly, with cross-cutting interconnections and influences. Those systems have been described as a Complexity Adaptative System (CAS).

CAS behavior is strongly determined by context. CAS self-organize with idiosyncratic and iterative interactions among the diverse stakeholder groups, accommodate behaviors and events, learn from experience, and dynamically evolve across time, giving rise to unpredictability and nonlinearity at the local level [17].

New system patterns (e.g., technology, policy, relationships, or practices) emerge through these interactions and exchanges. The main focus of implementation should not be to guarantee the fidelity of the intervention to be implemented but to leverage facilitators or eliminate barriers to tailoring and adapting the practice as required by context, harnessing the understanding and self-organizing capacity of local agents [18]. Paramount for the local adaptation of interventions is working with bottom-up local stakeholders facilitating ways to get them on board. LIWG in CHRODIS-PLUS had to address all these issues, adapting the existing knowledge regarding the practices and recommendations they were aiming to implement to their specific local contexts, providing feedback during the whole process to help embrace implementation iteratively over time.

Long-term sustainability is a further consideration of implementations. For improvement to have a lasting effect over time, a recursive process rather than a linear one should be expected [19], with the intervention evolving in an ongoing readaptation to the continuously transforming multilevel context. Whereas the early literature on sustainability focused largely on institutionalization (e.g., integrating the new set of practices into the routine operations of an organization), institutionalization may also become a barrier if it impedes institutions from adopting more effective practices when new evidence emerges. Sustainability has to be conceptualized as a dynamic construct that allows for retransformation in response to new or changing populations, evidence, or policies [20]. Sustainability requires paying attention not only to the context of an intervention but also to the interactions between elements and the consequences of this intervention for the system, adopting designs such as those foreseen by longer-term realist or process evaluation approaches [21].

## 5. Conclusions

The main lesson from the process of implementation of innovative practices and recommendations reported by CHRODIS-PLUS is the need to recognize the social dynamics associated with implementation, guaranteeing a sympathy toward the local culture and values that underpin these processes, which is a key differentiation from more linear improvement approaches [22].
